# Advanced Glycation End Product Blocker Drugs Have a Great Potential to Prevent Diabetic Cardiomyopathy in an Animal Model of Diabetes Mellitus Type-2

**DOI:** 10.1155/2022/7014680

**Published:** 2022-03-27

**Authors:** Amir Hossein Heydari, Mojtaba Fathi, Sophia Heydari, Mohammad Esmaeil Heidari

**Affiliations:** ^1^School of Medicine, Zanjan University of Medical Sciences, Zanjan, Zanjan, Iran; ^2^Biochemistry Department, Medical school, Qhazvin University of Medical Science, Qazvin, Qazvin, Iran; ^3^Shahid Beheshti Hospital, Zanjan University of Medical Sciences, Zanjan, Zanjan, Iran; ^4^Department of Electrical Engineering, Henry Samueli School of Engineering, University of California, Los Angeles, CA, USA

## Abstract

**Introduction:**

Sphingosine 1 phosphate (S1P) is a product of the sphingosine kinase 1 (SphK1) enzyme. Increased S1P can lead to tissue fibrosis that is also one of the pathways for developing diabetic cardiomyopathy. Advanced glycation end products (AGEs) increase S1P in cells. The study is aimed at using aminoguanidine (AG) as an AGEs blocker drug to prevent diabetic cardiomyopathy. *Materials and methods.* 210 rats were enrolled in the study. Diabetes mellitus type-2 was induced, and rats were divided into AG treated diabetic and nondiabetic groups. The heart histology was assessed with Masson's trichrome and hematoxylin-eosin staining. Cardiac function was measured with transthoracic echocardiography. S1P level and SphK1 gene expression were measured by western-blot and RT-qPCR, respectively.

**Results:**

Results showed that S1P level increases in diabetes, and its augmentation in cardiac tissue with K6PC-5 leads to cardiac fibrosis. 50 and 200 mg/kg of AG prevented cardiac fibrosis, but 100 mg/kg had no significant preventive effect. AG suppressed the SphK1 gene expression and reduced the fibrotic effect of S1P. AG preserved cardiac function by keeping ejection fraction and fractional shortening within the normal range in diabetic rats.

**Conclusion:**

AG has a suppressor effect on SphK1 gene expression besides its AGEs blocker role. AG is a potential drug to use in diabetic patients for preventing the development of diabetic cardiomyopathy. Other drugs that have AGEs or S1P blocker effects are a good choice for diabetic cardiomyopathy prevention.

## 1. Introduction

Cardiovascular disease (CVD) is the most important cause of morbidity and mortality in diabetes mellitus (DM) patients [1, 2]. Diabetic patients' chance of developing CVD is 2.5 folds more than nondiabetic patients [3, 4]. Diabetic cardiomyopathy (DCM) is defined as heart failure unrelated to hypertension and coronary artery diseases. The first manifestation of DCM is ventricular diastolic dysfunction, which has been attributed chiefly to cardiac fibrosis [5, 6].

Sphingosine kinase (SphK) is an enzyme that exists in most tissues. It has two subtypes, SphK1 phosphorylates sphingosine to sphingosine 1 phosphate (S1P) and SphK2 phosphorylate sphingosine to sphingosine 2 phosphate (S2P) [7]. SphK1 is an enhancer of cell proliferation and growth, and SphK2 mostly precludes apoptosis [8, 9]. Although S1P, the product of SphK1, can limit ischemia-reperfusion injury in the liver, brain, and kidney [10, 11], it has a significant role in tissue fibrosis. Advanced glycation end products (AGEs) are covalent components that are generated in long-term hyperglycemia. AGEs are one of the main fibrotic factors in organs, including the heart [12]. AGEs trigger the SphK1-S1P pathway that leads to tissue fibrosis [13].

Aminoguanidine (AG) is an inhibitor of nitric oxide synthase (iNOS) and blocker of AGEs, which has a cardioprotective role in the diabetic heart [14–16]. Since AG blocks AGEs in tissues, it is evident that AG decreases SphK1 activity through this pathway. K6PC-5 (N-(1,3-dihydroxyisopropyl)-2-hexyl-3-oxo-decanamide) is a recently developed potent SphK1 activator that has been studied in different diseases [17]. K6PC-5 is studied chiefly in bone, skin, and Ebola virus diseases.

The main aim of this study was to use of AG to show that inhibiting Sphk1 and reducing S1P can prevent diabetic cardiac fibrosis.

## 2. Methods

### 2.1. Animal Models and Study Design

For demonstrating the effect of increased S1P on cardiac fibrosis, the fibrosis area of the cardiac tissue was examined after the SphK1 gene enhancement. Then S1P level was measured in cardiomyocytes of diabetic rats to show that diabetes increases S1P levels in cardiomyocytes. In the last step, the hypothesis of the study was examined by inhibiting SphK1 with AG. All procedures were approved by the Zanjan University of Medical Science Animal Care and Use Committee with an approval number of A-10-141-6.

Ninety male *Rattus norvegicus* rats were enrolled in the study to show the S1P effect on cardiac fibrosis. All rats were 10-week-old. Rats were divided into two main groups, as control and K6PC-5 treated groups (K6PC-5). The control group was treated with daily saline as a placebo and the K6PC-5 group with daily 0.05 mg/kg of K6PC-5 (Creative Enzymes Co. Cat No. FLBZ-234). The K6PC-5 was daily injected intraperitoneally for 15 weeks. We injected the placebo in the same dose and interval as the K6PC-5.

The S1P level was assessed in cardiomyocytes of diabetic heart of forty 10-week-old male *Rattus norvegicus*. Rats were divided equally into two main groups. Twenty healthy rats were fed with a regular diet and no intervention (control group). Twenty rats were diabetized with streptozotocin (STZ) (diabetic group). Blood glucose of rats was measured by Accu-Chek Aviva Blood Glucose Monitoring device every seven days until the end of week 15. The caudal vein was used to measure blood glucose.

At last, we examined the central hypothesis of the study with eighty 10-week-old male *Rattus norvegicus* rats. Rats were equally assigned into two main groups, healthy and diabetic. Diabetes was induced with 40 mg/kg of streptozotocin, single intraperitoneal injection (STZ, Sigma-Aldrich Co.) [18]. Sigma-Aldrich aminoguanidine hydrochloride (AG, CAS Number 1937-19-5) was used to treat rats. Both groups were divided into four equal subgroups. All rats were matched in weight and age [15]. Healthy subgroups were healthy rats with high fat-high carbohydrate (HFHC) diet (CS group), healthy rats were treated with oral 50 mg/kg of AG plus HFHC diet (S50 group), healthy rats were treated with oral 100 mg/kg of AG plus HFHC diet (S100 group), and healthy rats were treated with oral 200 mg/kg of AG plus HFHC diet (S200 group). Diabetic subgroups were diabetic rats with HFHC diet (CD group), diabetic rats were treated with oral 50 mg/kg of AG plus HFHC diet (D50 group), diabetic rats were treated with oral 100 mg/kg of AG plus HFHC diet (D100 group), and diabetic rats were treated with oral 200 mg/kg of AG plus HFHC diet (D200 group).

### 2.2. Rat Cardiac Muscle and Blood Sample Preparation

Blood samples were obtained directly from the heart chamber at the end of the 15^th^ week. Rats were anesthetized with intraperitoneal xylazine and ketamine. We exterminated rats, according to the American Veterinary Medical Association. The heart was exposed with a left thoracotomy incision, and the free wall of the left ventricle's muscle was collected for further histological tests.

### 2.3. Cardiac Tissue Sample Masson's Trichrome and Hematoxylin-Eosin Staining

To assess cardiomyocytes and nucleus organization of ventricle samples, harvested rats' hearts were cut into multiple sections and embedded in paraffin and then stained with hematoxylin-eosin using standard methods [19]. We used a Video Analysis System (Jandel Scientific) to measure the length and the width of 50 myocytes in each sample and four samples for each rat to measure the cross-sectional area of myocytes (length x width). Paraffin-embedded samples were stained with Masson's trichrome using standard methods as a method to determine fibrosis area [20]. A blinded pathologist evaluated each sample, and the area of fibrosis was analyzed with AxioVision v.7 software.

### 2.4. S1P Level Measuring

S1P level was measured with the western blot method. Left ventricle tissue lysate and blood samples were serially diluted, and the protein concentration was measured with NanoDrop spectrophotometer (2 *μ*L of each sample was used). An equal amount of protein concentration of each sample was selected for further analysis. We measured 500 micrograms protein containing of each blood and tissue lysate samples by using the measured concentration of proteins in Nanodrop spectrophotometer and placed it on a nitrocellulose membrane (needed amount of sample (*μ*L) = 1000/concentration). Each sample was analyzed three times to ensure the results. The membranes were incubated with the anti-S1P antibody (Echelon Biosciences, Cat. Number: Z-P300). For reference, an anti-actin antibody (Sigma-Aldrich, Cat. Number: A5441) was used. Finally, spots were visualized on membranes by ECL plus and turned into quantitative numbers with Image Lab Software (Bio-Rad Laboratories).

### 2.5. Echocardiography

Transthoracic echocardiography (TTE) was conducted after anesthetizing rats with 100% oxygen and 1% isoflurane at the end of weeks 3, 9, and 15. TTE device was Philips SONOS 5500 system with a 12 MHz transducer. The ejection fraction and fractional shortening were measured at the mid-papillary slice.

### 2.6. Real-Time Quantitative PCR

Left ventricle samples' total mRNA was extracted. Extracted total mRNA of the samples was reverse transcriptased with PCR to synthesis cDNA. PCR amplified cDNA's number. Real time-qPCR was done with amplified cDNA samples, and the threshold cycles were calculated. Melting curves were used for purity confirmation. We normalized fluorescent signals with internal reference then set the threshold cycles (*C*_*T*_) for the increasing phase of the PCR. The Δ*C*_*T*_ method was used to show the results.  Table 1 shows the Primers construction.

### 2.7. Statistical Analysis

SPSS V.21 software was used to analyze data. Variance analyses (ANOVA) with Tukey post hoc tests were the main analytic method. GraphPad Prism was used for graphs (version 6.0). *p* < 0.05 indicates the significance of differences.

## 3. Results

### 3.1. General Specification of the Rats

The diabetic rats had significantly higher blood glucose, and they were heavier than nondiabetic rats. Heart weight in diabetic rats was significantly more than the healthy rats. Heart weight has been increased in K6PC-5 treated and decreased in AG treated diabetic rats. AG did not affect blood glucose, but it significantly decreased the heart weight of diabetic rats (Supplementary Table-1).

### 3.2. H&E Tissue Study

**Figure 1:**
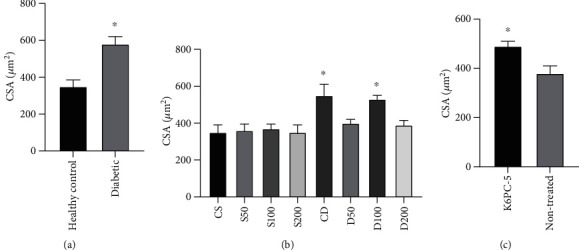
(a) Comparison of average of CSA between diabetic and healthy nontreated rats (^∗^*p* < 0.05). (b) Comparison of myocytes' cross-sectional area (^∗^*p* < 0.005). (c) Comparison of myocytes' cross-sectional area between K6PC-5 treated and nontreated rats (^∗^*p* < 0.05).

Cardiomyocyte CSA (cross-sectional area) was increased in diabetic rat, (Figure 1(a)) (supplementary Figure-1). AG significantly prevented the increase of cardiomyocyte CSA diabetic rats. In S50, S100, and S200 groups, AG treatment did not significantly affect the cardiomyocytes' CSA. Cardiomyocyte CSA in group D200 was significantly less than D100 and D50, and CSA of D50 was significantly less than D100 rats. Diabetes disturbed the architecture of the myocardium (increased CSA), and AG improved this disturbance (Figure 1(b)) (Supplementary Figure-2). K6PC-5 treated rats had significantly higher CSA than nontreated rats (Figure 1(c)) (Supplementary Figure-3).

### 3.3. Masson's Trichrome Tissue Study

**Figure 2:**
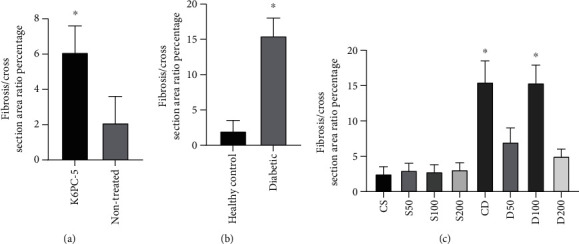
Percentage of fibrosis area/tissue crossarea ratio of studied rats. ^∗^*p* < 0.05, comparison of fibrosis percentage per cross-section area. Results are the average of 5 different tissue sections for each rat. (a) K6PC-5 rats have higher fibrosis tissue than nontreated rats. (b) Diabetic rats have higher fibrosis tissue than healthy control rats. (c) Pairwise comparison of different doses of aminoguanidine-treated diabetic rats, and CD and D100 rats have significantly more fibrotic cardiac tissue than other groups.

Fibrosis in cardiac tissue was prominent in K6PC-5 treated rats (supplementary Figure-4). Percentage of fibrosis area/tissue crossarea ratio compared between groups showed significantly higher fibrosis in K6PC-5 treated rats' cardiac tissue (Figure 2(a)). The fibrosis area/tissue crossarea ratio in diabetic rats was significantly higher than in nondiabetic rats (Figure 2(a)) (Supplementary Figure-5). Diabetic rats treated with 50 mg/kg and 200 mg/kg of the AG had less cardiac tissue fibrosis than nontreated and 100 mg/kg of AG treated diabetic rats (Supplementary Figure-6). % fibrosis area/tissue crossarea ratio was significantly higher in CD and D100 cardiac tissue than all other groups. There was no significant difference in the % fibrosis area/tissue crossarea ratio between D50, D200, CS, S50, S100, and S200 groups (Figure 2(c)).

### 3.4. S1P Level

**Figure 3:**
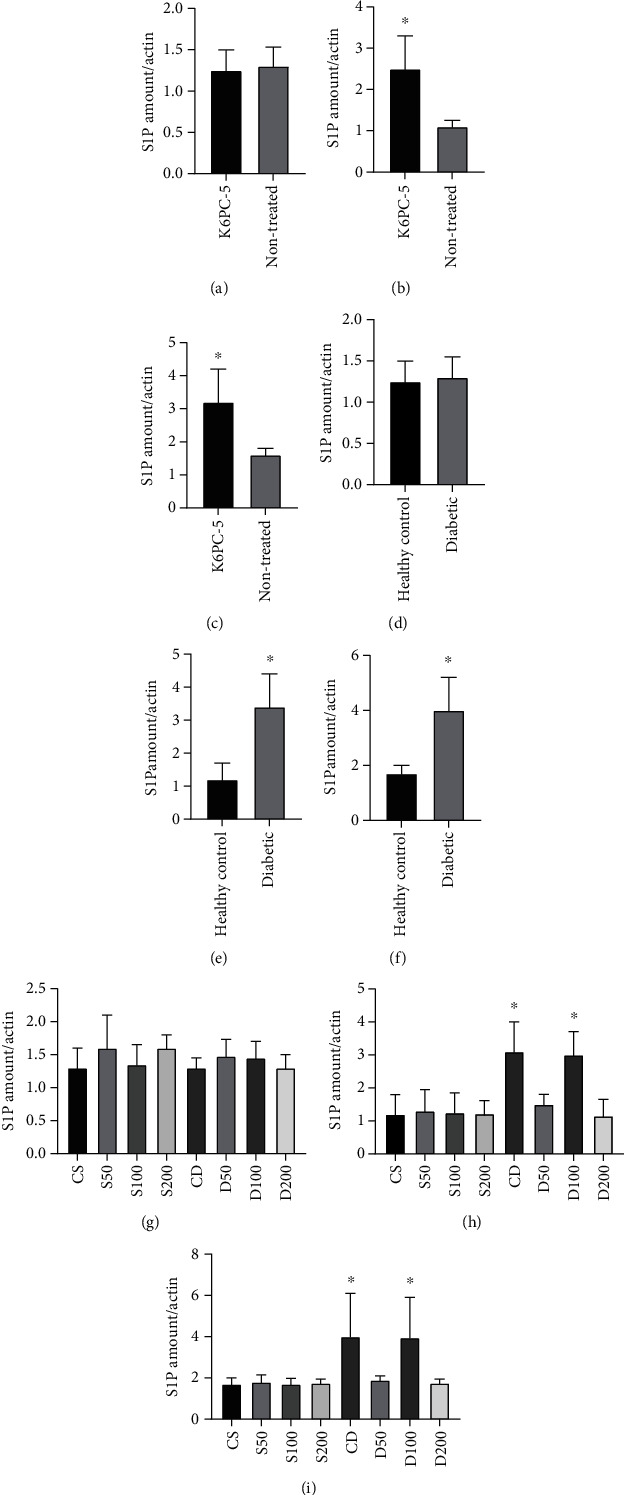
(a) Comparison of the first day S1P level of blood of K6PC-5 treated and nontreated rats. (b) Comparison of last day S1P level of blood of K6PC-5 treated and nontreated rats (^∗^*p* < 0.05). (c) Comparison of last day S1P level of cardiac tissue of K6PC-5 treated and nontreated rats (^∗^*p* < 0.05). (d) Comparison of first day S1P level of blood of healthy and diabetic rats. (e) Comparison of last day S1P level of blood of healthy and diabetic rats (^∗^*p* < 0.05). (f) Comparison of last day S1P level of cardiac tissue of healthy and diabetic rats (^∗^*p* < 0.05). (g) Comparison of blood samples' S1P level of the first day of CS, S50, S100, S200, CD, D50, D100, and D200 groups. (h) Comparison of blood samples' S1P level of last day of CS, S50, S100, S200, CD, D50, D100, and D200 groups (^∗^*p* < 0.001). (i) Comparison of cardiac tissue samples' S1P level of last day of CS, S50, S100, S200, CD, D50, D100, and D200 groups (^∗^*p* < 0.001).

S1P levels of the blood and tissue samples of K6PC-5 treated rats were significantly higher than nontreated rats (supplementary Figure-7). S1P levels of the blood and tissue samples of diabetic rats were significantly higher than diabetic rats (supplementary Figure-8). S1P level of blood and tissue samples of CD and D100 rats was significantly more than healthy rats. S1P levels of D50 and D200 were significantly less than CD and D100 (Supplementary Figure-9) (Figure 3).

### 3.5. Real-Time Quantitative PCR Results

**Figure 4:**
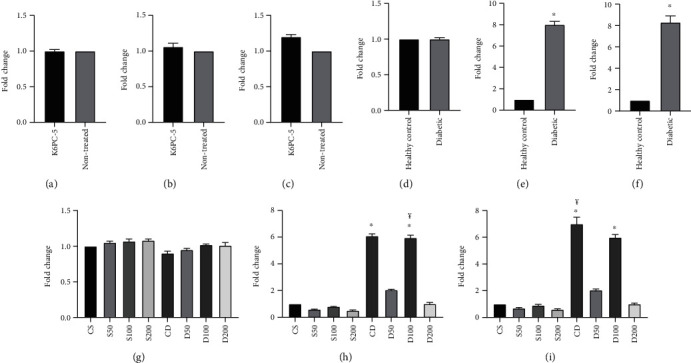
(a) Comparison of first day's fold changes of blood samples of groups, considering nontreated group equal to 1 (other group is shown proportional to the nontreated group). (b) Comparison of last day's fold changes of blood samples of groups, considering nontreated group equal to 1 (other group is shown proportional to nontreated group, ^∗^*p* < 0.05). (c) Comparison of last day's fold changes of cardiac tissue samples of groups, considering nontreated group equal to 1 (other group is shown proportional to nontreated group, ^∗^*p* < 0.05). (d) Comparison of first day's fold changes of blood samples of groups, considering healthy group equal to 1 (other group is shown proportional to the healthy group). (e) Comparison of last day's fold changes of blood samples of groups, considering healthy group equal to 1 (other group is shown proportional to the healthy group, ^∗^*p* < 0.001). (f) Comparison of last day's fold changes of cardiac tissue samples of groups, considering the healthy group equal to 1 (other group is shown proportional to the healthy group, ^∗^*p* < 0.001). (g) Comparison of first day's fold changes of blood samples of groups, considering the CS group equal to 1 (other group is shown proportional to CS). (h) Comparison of last day's fold changes of blood samples of groups, considering the CS group equal to 1 (other group is shown proportional to CS, ^∗^*p* < 0.001, ¥ D50 vs. D200 *p* < 0.05). (i) Comparison of last day's fold changes of cardiac tissue samples of groups, considering the CS group equal to 1 (other group is shown proportional to CS, ^∗^*p* < 0.001, ¥ D50 vs. D200, *p* < 0.05).

K6PC-5 group's *SphK1* gene expression was not significantly different from the healthy nontreated group (supplementary table-2). The *SphK1* gene expression was significantly higher in non-treated diabetic rats than healthy control rats (supplementary table-3). RT-qPCR of ventricle and blood samples showed significantly higher *SphK1* gene expression in CD and D100 than other groups. The *SphK1* gene expression was not significantly different between D50, D200, CS, S50, S100, and S200 groups. There was no significant difference in the *SphK1* gene expression between D100, S100, CD, and CS groups (supplementary table-4) (Figure 4). Fold changes were calculated by 2^−ΔΔ*Ct*^: Δ*Ct* = *Ct*_Sphk1_ − *Ct*_hprt_.ΔΔ*Ct* = Δ*Ct*_AG treated_ − Δ*Ct*_CS_.

### 3.6. Echocardiography

**Figure 5:**
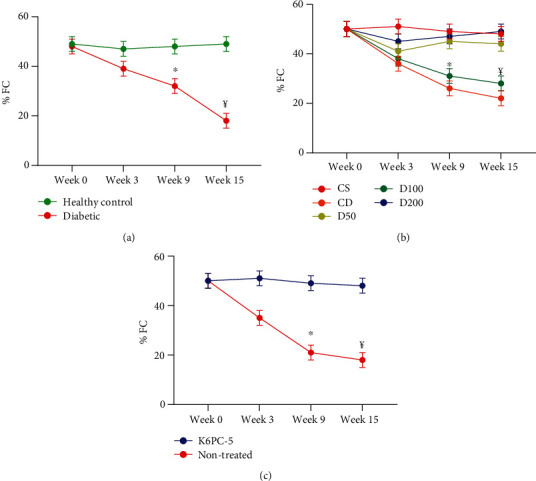
Mean EF and FC of studied groups throughout the experiment. (a) Healthy and diabetic groups. (b) CS, S50, S100, S200, CD, D50, D100, and D200 groups. (c) K6PC-5 and nontreated groups. ^∗^*p* < 0.05, ¥*p* < 0.001.

Mid papillary short axis-2D transthoracic echocardiography showed a significant decrease of ejection fraction (%EF) and fractional shortening (%FS) from the 3^rd^ week to the end of the study in K6PC-5 rats (supplementary table-5) (Figure 5).

Mid papillary short axis-2D transthoracic echocardiography showed a significant decrease of ejection fraction (%EF) and fractional shortening (%FS) from the 3^rd^ week to the end of the study in diabetic rats (supplementary table-6) (Figure 5).

Echocardiography showed that a decrease of EF and FS in diabetic rats had been begun since the 9^th^ week after rats was made diabetic. EF and FS in D50 and D200 rats started to improve since week 9. There was a significant difference in EF and FS in CD and D-100 groups with all other groups since the 9^th^ week (Supplementary table-7) (Figure 5).

## 4. Discussion

Injection of K6PC-5 to rats increased S1P level in cardiomyocytes and heart blood of the rats. These rats had significantly higher fibrotic tissue in their hearts than the rats that had normal S1P levels. Increasing fibrotic tissue proportion to normal tissue in rats with increased S1P level led to a progressive decrease in heart function (decreased %EF and %FC). These findings show that increasing only the S1P level with an S1P specific drug such as K6PC-5 can induce fibrosis and hypertrophy in cardiac tissue. S1P level in blood and cardiac tissue is directly associated with the extension of the cardiac tissue fibrosis area. We detected a high level of S1P in the blood and cardiac tissue of the diabetic rats. This increment of S1P was associated with increasing the expression of the SphK1's gene in cardiomyocytes. Diabetes increased the production of S1P by increasing the expression of the SphK1 gene. As earlier, our study showed that a pure increase of S1P can lead to cardiac fibrosis; it can be concluded that one of the primary cardiac fibrotic pathways in diabetes is associated with SphK1 gene expression alteration (supplementary figure-10). Recent studies showed that aminoguanidine is a blocker of AGEs in cells, and these AGEs are responsible for increasing the S1P level. Aminoguanidine was able to decrease the S1P level in diabetic rats by this pathway. In addition to limiting the AGEs from increasing the S1P level, AG was also able to decrease the SphK1 gene expression; these two pathways seem to be the chief route of anti-S1P specification of AG. AG inhibited the cardiac tissue from developing fibrotic scars and cardiomyocytes hypertrophy (measured by CSA) in diabetic rats. This inhibition helped diabetic rats to maintain their cardiac function as measured by %EF and %FC. We expected that higher doses of AG would have more significant effects, but despite 50 and 200 mg/kg of AG, 100 mg/kg of AG did not show any effective antifibrotic action in diabetic rats. The primary outcome of decreasing S1P was that the fibrotic area remained limited in diabetic rat's hearts; so, the cardiac function (%EF and %FC) remained within the normal range.

Ikeda and et al. showed that S1P deficient mice had a slow liver fibrosis process. They discussed that lack of S1P is a crucial factor that inhibits the fibrosis process [21] (supplementary figure-11). Shea et al. induced lung injury by administrating bleomycin in mice. They showed that prolonged exposure to S1P agonists exacerbates the lung injury and fibrosis process [22]. Lowe et al. experimented S1P effects on myofibroblast of mice. They concluded that TGF-*β* activated the S1P-SphK1 pathway in cardiac fibroblasts and enhances collagen production. They assessed this pathway on cell line models [23]. Ohkura and et al. showed that S1P receptors are enhanced by angiotensin-II, which leads to cardiac muscle hypertrophy and fibrosis [24]. All mentioned studies, especially those that examined the cardiac tissue, did not discuss S1P effects in the diabetic heart. There are few studies about S1P roles in cardiac tissue pathologies, but there is no study about manipulating S1P level and gene expression. In this study, we changed the activity of the S1P pathway with K6PC-5. We did this intervention on an animal that is alive and examined it in vivo, which makes our study a unique experiment in this field. We showed that increasing the S1P level and activity by K6PC-5 increases cardiac fibrosis and decreases cardiac function. None of the previous studies examined cardiac function with TEE. We also showed that decreasing SphK1, the source of the S1P, with aminoguanidine could be a novel approach to preventing diabetic cardiomyopathy. Our study showed the positive protective effect of AG on diabetic rats by measuring cardiac function with TTE. Yildirim et al. and Giri et al. showed that AG could be used to ameliorate lung fibrosis in mice treated with bleomycin [25, 26]. Parthasarathy et al. showed that AG is capable of reducing isoproterenol-induced cardiac hypertrophy and fibrosis. They used 50 mg/kg of AG in their study [27].

The most similar study to ours is an article by Magdaleno et al. They used 20 mg/kg AG in diabetic rats to assess the effects of this drug on cardiac fibrosis. They showed that AG inhibits cardiac fibrosis by ERK1/2 and SMAD2/3 pathways (supplementary data figure-10 and Figure 11) [15]. Our study's privilege to their study is that we practically showed the positive effects of AG on cardiac function by TTE. We also proposed a new pathway for antifibrotic effects of the AG through S1P-SphK1. Based on the S1P-SphK1 pathway role in diabetic cardiac fibrosis, many experimental and approved drugs that exert their effects by S1P or AGE inhibition pathways can be considered instead of AG. We also titrated the dose of AG to show the dose effect of this drug on cardiac fibrosis. Our study succeeded in almost fully preventing the heart from developing fibrotic tissue in diabetic rats.

However, Magdaleno and his colleagues just reduced the fibrosis (they could not stop the fibrosis process). The cardioprotective role of AG is strongly associated with the SphK1 gene expression and increasing the S1P level in the diabetic heart. One of the most critical factors that limit this effect is the dose of AG. 100 mg/kg of AG was not effective as much as 50 and 200 mg/kg, which seems to be because of the compensatory response mechanism. When a drug enters the bloodstream, body cells activate a regulator process and adapt regulator. Target cells detect drug substances as foreign materials; so, they counteract the drug [28]. These regulatory processes are activated by a particular dose of the drug that is drug type-dependent. Doses lower than the activator dose do not activate the regulatory processes so that they can exert their effects, like 50 mg/kg of AG in this study. This compensatory response has enough power to neutralize drug effects at a specific dose as in the D100 group. When 100 mg/kg of AG was used in healthy rats, the compensatory response neutralized AG, and its adverse mechanism caused an inhibitory effect on the SphK1 expression (see supplementary data axillary section and supplementary figure-12). 200 mg/kg of AG was more effective because it is more than the compensatory response's power to neutralize it. 200 mg/kg of AG can escape the counteracting effect of compensatory response and exerts its effects. K6PC-5 treating study showed that S1P alone could be the most important fibrotic agent in cardiac fibrosis.

## 5. Conclusion

This study showed that using drugs that block AGEs or S1P function in the heart can prevent the diabetic heart from producing fibrotic dead tissue. Our study showed that the S1P-SphK1 pathway is one of the prominent role players of diabetic cardiac fibrosis; thus, suppressing the SphK1 gene expression can be considered a potential means to prevent diabetic cardiomyopathy. Our study revealed a new route for AG's anti-fibrotic effect by affecting the S1P-SphK1 pathway. This study showed that AG could prevent diabetic cardiomyopathy and preserve cardiac function in diabetic conditions. We recommend that scientists do the same study as ours on human models.

There are some limitations in our study that we faced during the experiment. The first and most important limitation was that we could not combine AG with other antidiabetic assessed their probable interactions because of the financial limitations. Another limitation of our study was that we did not assess the laboratorial side effects of AG; although, there were no discernible external side effects. Last but not least limitation of our study was that we had three rats that died from an infection in AG-treated diabetic groups, but we were not able to discriminate that these deaths were due to AG or diabetes.

### 5.1. Research in Context

What are already known in this context?

- S1P plays a pivotal role in fibrosis development in most organs such as the lung, kidney, retina, and heart
- Aminoguanidine has an ant-fibrotic effect in diabetes through ERK1/2 and SMAD2/3 pathways

What is the main question?

- Does aminoguanidine exert its antifibrotic effect by S1P-SphK1 pathways and can it entirely prevent deterioration of the cardiac function in diabetes?

What are the new findings?

- S1P alone is responsible for cardiac fibrosis in diabetes type-2
- AGE inhibitor drugs such as aminoguanidine can prevent diabetic cardiomyopathy by decreasing S1P activity and production
- AG suppresses the SphK1 gene expression and S1P production in cardiac tissue

What are the impacts on clinical practice?

AGE blocker drugs can decrease S1P production; so, they can be considered the primary approach to preventing diabetic cardiomyopathy in diabetes mellitus type-2; one of these drugs is aminoguanidine

## Figures and Tables

**Table 1:** Hypoxanthine–guanine phosphoribosyltransferase (hprt), housekeeping, and target gene primer sequence. SphK: sphingosine kinase.

Gene	Primer sequence (5′-3′)	Amplicon size (bp)
*SphK1*	F: CTTCCTTGAACCATTATGCTG R: GCCGATACTTCTCACTCTC	204
*Hprt*	F: AAGCTTGCTGGTGAAAAGGA R: TTGCGCTCATCTTAGGCTTT	198

## Data Availability

Data will be available if asked from the corresponding author E-mail: amhohe@gmail.com.

## Abbreviations

CVD: Cardiovascular disease
DM: Diabetes mellitus
DCM: Diabetic cardiomyopathy
PKC: Protein kinase C
ROS: Reactive oxygen species
TAG: Triacylglycerol
TGF-*β*: Transforming growth factor-beta
SphK1: Sphingosine kinase-1
S1P: Sphingosine 1 phosphate
HDAC2: Histone deacetylase-2
AG: Aminoguanidine
iNOS: Inhibitor of nitric oxide synthase
AGEs: Advanced glycation end products
H&E: Hematoxylin-eosin
CS: Healthy rats with no treatment
S50: Healthy rats treated with 50 mg/kg AG
S100: Healthy rats treated with 100 mg/kg AG
S200: Healthy rats treated with 200 mg/kg AG
CD: Diabetic rats with no treatment
D50: Diabetic rats treated with 50 mg/kg AG
D100: Diabetic rats treated with 100 mg/kg AG
D200: Diabetic rats treated with 200 mg/kg AG
% EF: % ejection fraction
% FS: % fractional shortening.

## Supplementary Materials

Supplementary Materials Supplementary Table 1: demographic information of studied rats. Ɨ*p* < 0.05 diabetic compared to healthy rats; ^∗^*p* < 0.05 comparison between CS, S50, S100, S200, CD, D50, D100, and D200. ¥*p* < 0.05 comparison between K6PC-5 treated and nontreated rats. Supplementary Table 2: fold changes of cardiac ventricle tissue and blood samples of K6PC-5 treated and nontreated rats (hprt gene as housekeeping), considering nontreated rats equal to 1 (the K6PC-5 treated group is proportional to nontreated rats). Supplementary Table 3: fold changes of cardiac ventricle tissue and blood samples of diabetic and healthy rats (hprt gene as housekeeping), considering healthy rats equal to 1 (the diabetic-treated group is proportional to healthy rats, ^∗^*p* < 0.001). Supplementary Table 4: fold changes of ventricle and blood samples of groups (hprt gene as housekeeping), considering CS equal to 1 (other groups are shown proportional to CS). CS: healthy W/O AG; S50: healthy with 50 mg/kg AG; S100: healthy with 100 mg/kg AG; S200: healthy with 200 mg/kg AG; CD: diabetic W/O AG; D50: diabetic with 50 mg/kg AG; D100: diabetic with 100 mg/kg AG; D200: diabetic with 200 mg/kg AG (^∗^*p* < 0.001). Comparison of D50 and D200 shows significantly higher SphK1 expression in D50 (¥*p* < 0.05). Supplementary Table 5: % ejection fraction (%EF) and % fractional shortening (%FC) comparison between K6PC-5 treated and nontreated groups. ^∗^*p* < 0.05, Ɨ*p* < 0.001. Supplementary Table 6: % ejection fraction (%EF) and % fractional shortening (%FC) comparison between healthy and diabetic groups. ^∗^*p* < 0.05, ¥*p* < 0.001. Supplementary Table 7: % ejection fraction (%EF) and % fractional shortening (%FC) comparison between groups at phase II. ^∗^*p* < 0.05, Ɨ*p* < 0.05 (all *p* values are <0.05 for week 9 and <0.001 for week 15). Supplementary Figure 1: this image of the H&E stained section (×400) of diabetic rat (a) shows architecture disturbance in the field compared to healthy nontreated rat (b). Supplementary Figure 2: image of H&E stained section (×100) of CD (a), CS (b), D50 (c), S50 (d), D100 (e), S100 (f), D200 (g), and S200 (h). Cardiomyocyte arrangement was altered in CD and D50, but in D50 and D200, it is almost the same as nondiabetic cardiomyocytes. CS: healthy W/O AG; S50: healthy with 50 mg/kg AG; S100: healthy with 100 mg/kg AG; S200: healthy with 200 mg/kg AG; CD: diabetic W/O AG; D50: diabetic with 50 mg/kg AG; D100: diabetic with 100 mg/kg AG; D200: diabetic with 200 mg/kg AG. Supplementary Figure 3: image of H&E stained section (×100) of healthy control rats (a) and K6PC-5 treated (b). Cardiomyocyte CSA was increased in K6PC-5-treated rats (×100 amplification). Supplementary Figure 4: Masson's trichrome stained cardiac sections of nontreated rats (a) vs. K6PC-5-treated rats (b). Fibrosis can be seen in cardiac tissue of K6PC-5-treated rats (×40 amplification). Supplementary Figure 5: Masson's trichrome stained sections (×100) of healthy control rats (a) and diabetic rats (b). Significant decreased fibrosis is seen in diabetic rats.
