# Supplementary material for: Advanced Glycation End Product Blocker Drugs Have a Great Potential to Prevent Diabetic Cardiomyopathy in an Animal Model of Diabetes Mellitus Type-2
Source: Cardiovasc Ther. 2022 Mar 27;2022:7014680. doi: 10.1155/2022/7014680 (PMC8977315; doi:10.1155/2022/7014680)
Supplement: Supplementary Materials — Supplementary Table 1: demographic information of studied rats. Ɨp < 0.05 diabetic compared to healthy rats; ∗p < 0.05 comparison between CS, S50, S100, S200, CD, D50, D100, and D200. ¥p < 0.05 comparison between K6PC-5 treated and nontreated rats. Supplementary Table 2: fold changes of cardiac ventricle tissue and blood samples of K6PC-5 treated and nontreated rats (hprt gene as housekeeping), considering nontreated rats equal to 1 (the K6PC-5 treated group is proportional to nontreated rats). Supplementary Table 3: fold changes of cardiac ventricle tissue and blood samples of diabetic and healthy rats (hprt gene as housekeeping), considering healthy rats equal to 1 (the diabetic-treated group is proportional to healthy rats, ∗p < 0.001). Supplementary Table 4: fold changes of ventricle and blood samples of groups (hprt gene as housekeeping), considering CS equal to 1 (other groups are shown proportional to CS). CS: healthy W/O AG; S50: healthy with 50 mg/kg AG; S100: healthy with 100 mg/kg AG; S200: healthy with 200 mg/kg AG; CD: diabetic W/O AG; D50: diabetic with 50 mg/kg AG; D100: diabetic with 100 mg/kg AG; D200: diabetic with 200 mg/kg AG (∗p < 0.001). Comparison of D50 and D200 shows significantly higher SphK1 expression in D50 (¥p < 0.05). Supplementary Table 5: % ejection fraction (%EF) and % fractional shortening (%FC) comparison between K6PC-5 treated and nontreated groups. ∗p < 0.05, Ɨp < 0.001. Supplementary Table 6: % ejection fraction (%EF) and % fractional shortening (%FC) comparison between healthy and diabetic groups. ∗p < 0.05, ¥p < 0.001. Supplementary Table 7: % ejection fraction (%EF) and % fractional shortening (%FC) comparison between groups at phase II. ∗p < 0.05, Ɨp < 0.05 (all p values are <0.05 for week 9 and <0.001 for week 15). Supplementary Figure 1: this image of the H&E stained section (×400) of diabetic rat (a) shows architecture disturbance in the field compared to healthy nontreated rat (b). Supplementary Figure 2: image of H&E s [file 7014680.f1.docx]

*Supplementary table 1*

|  |  | healthy | diabetic | CS | S50 | S100 | S200 | CD | D50 | D100 | D200 | Non-treated | K6PC-5 |
| --- | --- | --- | --- | --- | --- | --- | --- | --- | --- | --- | --- | --- | --- |
| Weight (g) | 1^st^ day | 221±15 | 223±21 | 224±8 | 221±8 | 224±7 | 223±7 | 224±6 | 224±7 | 223±7 | 223±8 | 223±8 | 225±3 |
|  | Last day | 442±23 | 560±32^Ɨ^ | 452±12 | 458±15 | 451±10 | 456±9 | 549±15^*^ | 541±10^*^ | 548±30^*^ | 539±16^*^ | 410±30 | 391±29 |
| Heart weight (mg) | Last day | 132±2 | 161±4^Ɨ^ | 135±2 | 136±1 | 135±4 | 134±2 | 158±5^*^ | 139±2 | 156±1^*^ | 136±3 | 132±3 | 151±3^¥^ |
| Food intake (g/24h) | 1^st^ day | 15.6±1.3 | 15.5±1.5 | 15.8±1.3 | 15.5±1.3 | 16±1.3 | 16.6±1.5 | 15.6±1.2 | 16±1.1 | 16±1.4 | 16.2±1.2 | 15.7±1.5 | 14.9±3.2 |
|  | Last day | 24±2 | 30±1^Ɨ^ | 26±1 | 26±4 | 25±2 | 27±2 | 32±2^*^ | 31±1^*^ | 31±2^*^ | 33±4^*^ | 25±4 | 22±6 |
| Blood glucose (mg/dl) | 1^st^ day | 109±11 | 112±10 | 110±10 | 108±11 | 106±12 | 110±9 | 107±10 | 109±11 | 108±11 | 107±12 | 110±12 | 106±12 |
|  | Last day | 108±15 | 356±21^Ɨ^ | 123±10 | 126±5 | 131±12 | 128±8 | 294±30^*^ | 302±26^*^ | 301±29^*^ | 292±14^*^ | 109±9 | 110±34 |

Supplementary Table 1, Demographic information of studied rats. Ɨ p<0.05 diabetic compared to healthy rats; *p<0.05 comparison between CS, S50, S100, S200, CD, D50, D100, and D200. ¥ p<0.05 comparison between K6PC-5 treated and non-treated rats.

Supplementary Table 2

| Groups | | K6PC-5 | Non-treated |
| --- | --- | --- | --- |
| Blood | Fold change (first day) | 1.02±0.02 | 1 |
|  | Fold change (last day) | 1.11±0.04 | 1 |
| Cardiac tissue | Fold change (last day) | 1.22±0.03 | 1 |

Supplementary Table 2. Fold changes of cardiac ventricle tissue and blood samples of K6PC-5 treated and non-treated rats (*hprt* gene as housekeeping), considering non-treated rats equal to 1 (K6PC-5 treated group is proportional to non-treated rats)

Supplementary Table 3

| Groups | | Diabetic | Healthy |
| --- | --- | --- | --- |
| Blood | Fold change (first day) | 1.01±0.02 | 1 |
|  | Fold change (last day) | 8.12±0.34* | 1 |
| Cardiac tissue | Fold change (last day) | 8.65±0.53* | 1 |

Supplementary Table 3. Fold changes of cardiac ventricle tissue and blood samples of diabetic and healthy rats (*hprt* gene as housekeeping), considering healthy rats equal to 1 (diabetic treated group is proportional to healthy rats, * p<0.001)

Supplementary Table 4

| Groups | | CS | S50 | S100 | S200 | CD | D50 | D100 | D200 |
| --- | --- | --- | --- | --- | --- | --- | --- | --- | --- |
| Blood | Fold change (first day) | 1 | 1.02 ±0.02 | 1.03±0.03 | 1.04±0.02 | 0.98±0.04 | 0.99±0.03 | 1.02±0.02 | 1.01±0.06 |
|  | Fold change (last day) | 1 | 0.61±0.01 | 0.83±0.03 | 0.48±0.02 | 6.33±0.31* | 2.12±0.09^¥^ | 5.91±0.21* | 1.02±0.11^¥^ |
| Ventricle | Fold change (last day) | 1 | 0.72±0.02 | 0.85±0.04 | 0.55±0.02 | 6.99±0.52* | 2.15±0.08 ^¥^ | 6.02±0.23* | 1.04±0.08 ^¥^ |

Supplementary Table 4. Fold changes of ventricle and blood samples of groups (*hprt* gene as housekeeping), considering CS equal to 1 (other groups are shown proportional to CS). CS: healthy W/O AG, S50: healthy with 50 mg/kg AG, S100: healthy with 100 mg/kg AG, S200: healthy with 200 mg/kg AG, CD: diabetic W/O AG, D50: diabetic with 50 mg/kg AG, D100: diabetic with 100 mg/kg AG, D200: diabetic with 200 mg/kg AG (* p<0.001). Comparison of D50 and D200 shows significantly higher SphK1 expression in D50 (¥ p<0.05)

Supplementary Table 5

|  | Non-treated | | K6PC-5 | |
| --- | --- | --- | --- | --- |
|  | %EF | %FC | %EF | %FC |
| WEEK 0 | 73±1 | 50±2 | 73±1 | 51±3 |
| WEEK 3 | 74±1 | 51±2 | 60±1 | 35±2 |
| WEEK 9 | 75±2 | 49±1 | 44±1* | 21±3* |
| WEEK 15 | 74±3 | 48±3 | 29±2^¥^ | 15±5^¥^ |

Supplementary Table 5; % ejection fraction (%EF) and %fractional shortening (%FC) comparison between K6PC-5 treated and non-treated groups. *p<0.05, Ɨ p<0.001

Supplementary Table 6

|  | Healthy | | Diabetic | |
| --- | --- | --- | --- | --- |
|  | %EF | %FC | %EF | %FC |
| WEEK 0 | 72±2 | 50±1 | 71±1 | 49±2 |
| WEEK 3 | 73±1 | 49±2 | 61±3 | 39±1 |
| WEEK 9 | 75±1* | 50±2* | 46±3* | 33±2* |
| WEEK 15 | 76±2^¥^ | 51±1 ^¥^ | 25±3^¥^ | 16±2 ^¥^ |

Supplementary Table 6; % ejection fraction (%EF) and %fractional shortening (%FC) comparison between healthy and diabetic groups. *p<0.05, ¥ p<0.001.

Supplementary Table 7

|  | CS | | S50 | | S100 | | S200 | | CD | | D50 | | D100 | | D200 | | |
| --- | --- | --- | --- | --- | --- | --- | --- | --- | --- | --- | --- | --- | --- | --- | --- | --- | --- |
|  | %EF | %FC | %EF | %FC | %EF | %FC | %EF | %FC | %EF | %FC | %EF | %FC | %EF | %FC | %EF | %FC |  |
| WEEK 0 | 73±2 | 51±2 | 73±1 | 51±1 | 72±2 | 51±3 | 73±1 | 53±1 | 72±2 | 52±4 | 73±2 | 51±3 | 71±3 | 52±2 | 72±2 | 52±2 |  |
| WEEK 3 | 73±1 | 52±2 | 72±2 | 50±3 | 72±1 | 52±2 | 73±2 | 52±2 | 62±2 | 38±2 | 62±1 | 42±2 | 62±3 | 40±1 | 63±2 | 43±3 |  |
| WEEK 9 | 74±2 | 51±1 | 72±3 | 50±2 | 73±1 | 52±3 | 73±2 | 52±3 | 48±3* | 28±1^Ɨ^ | 65±2 | 46±2 | 50±2* | 31±3 ^Ɨ^ | 68±2 | 44±3 |  |
| WEEK 15 | 73±3 | 50±3 | 73±1 | 51±1 | 72±2 | 52±1 | 72±1 | 52±4 | 31±2* | 21±2 ^Ɨ^ | 68±3 | 46±3 | 42±2* | 29±2 ^Ɨ^ | 71±2 | 48±2 |  |

Supplementary Table 7; % ejection fraction (%EF) and %fractional shortening (%FC) comparison between groups at phase II. *p<0.05, Ɨ p<0.05 (all p values are <0.05 for week 9 and <0.001 for week 15)

***Figures***


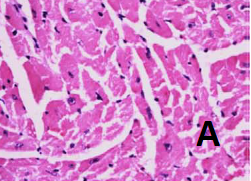

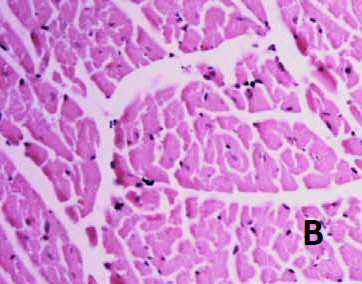


2µm

Supplementary figure 1; This image of the H&E stained section (400x) of diabetic rat (A) shows architecture disturbance in the field compared to healthy non-treated rat (B).


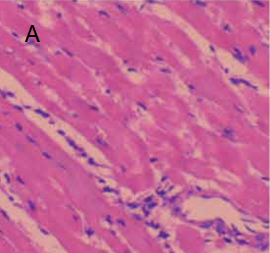

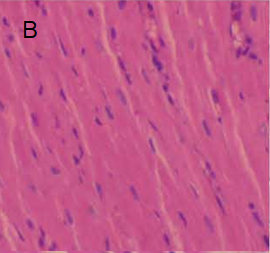

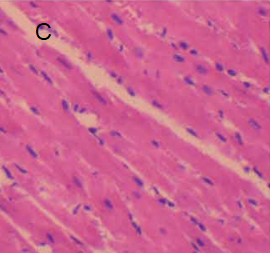

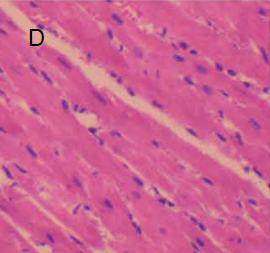

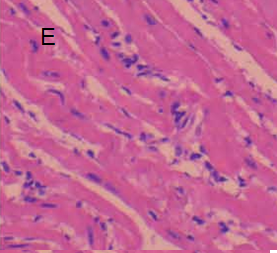

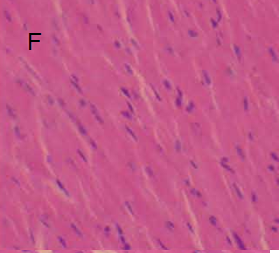

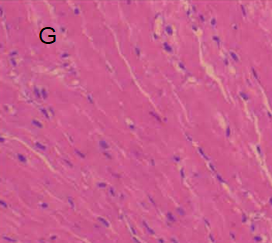

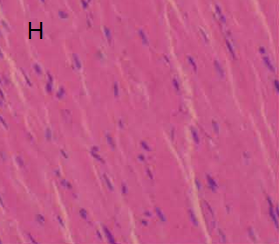


5µm

Supplementary figure 2; image of H&E stained section (100x) of CD (A), CS (B), D50 (C), S50 (D), D100 (E), S100 (F), D200 (G), and S200 (H). Cardiomyocytes arrangement was altered in CD and D50, but in D50 and D200 it is almost the same as non-diabetic cardiomyocytes. CS: healthy W/O AG, S50: healthy with 50 mg/kg AG, S100: healthy with 100 mg/kg AG, S200: healthy with 200 mg/kg AG, CD: diabetic W/O AG, D50: diabetic with 50 mg/kg AG, D100: diabetic with 100 mg/kg AG, D200: diabetic with 200 mg/kg AG;


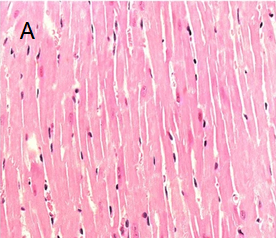

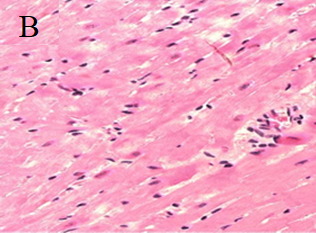


5 µm

Supplementary figure 3; image of H&E stained section (100x) of healthy control rats (A), K6PC-5 treated (B). Cardiomyocytes CSA was increased in K6PC-5 treated rats (x100 amplification).


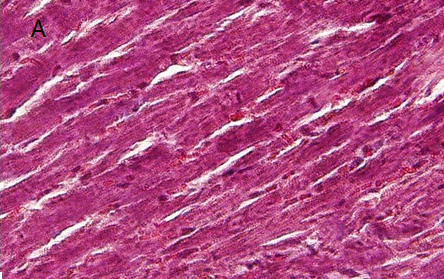

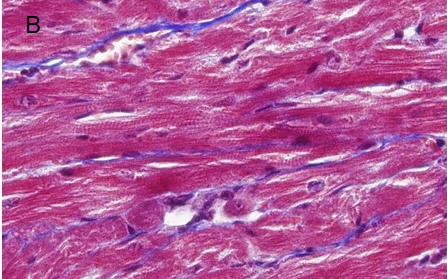


2 µm

Supplementary figure 4; Masson's trichrome stained cardiac sections of non-treated rats (A) vs. K6PC-5 treated rats (B). Fibrosis can be seen in cardiac tissue of K6PC-5 treated rats (x40 amplification).


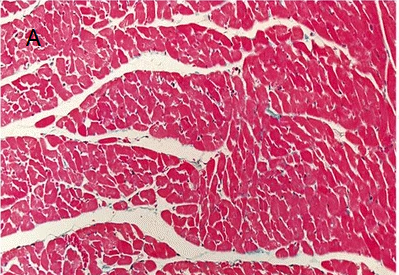

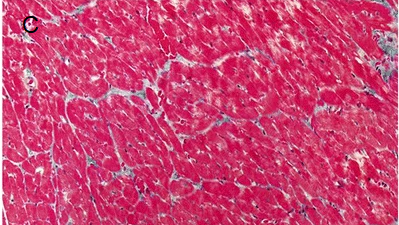


A

B

5µm

Supplementary figure 5; Masson's trichrome stained sections (100x) of Healthy control rats (A), diabetic rats (B). Significant decreased fibrosis is seen in diabetic rats.


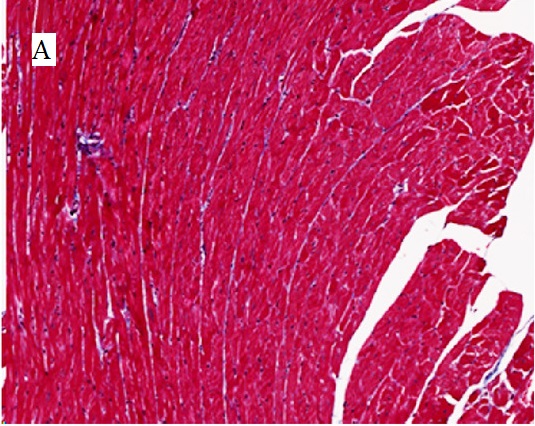

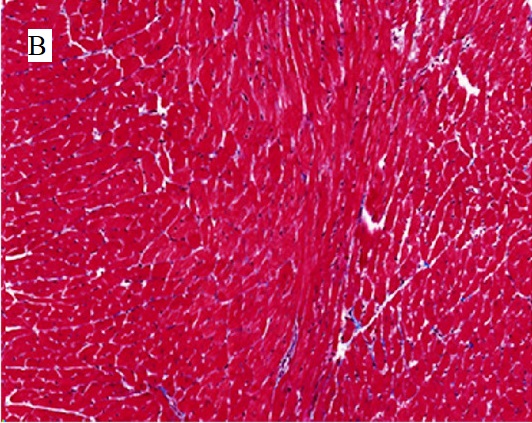

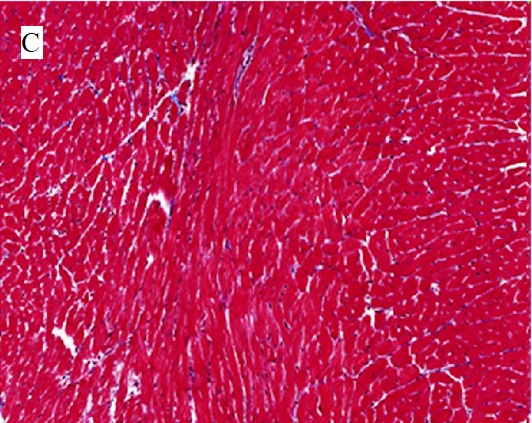

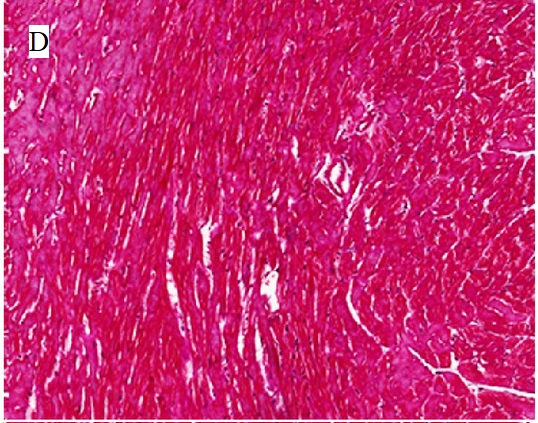

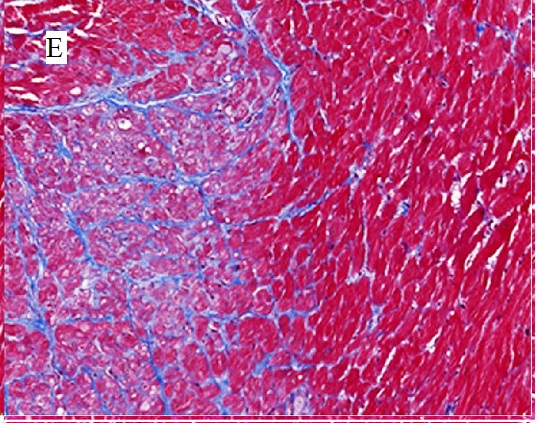

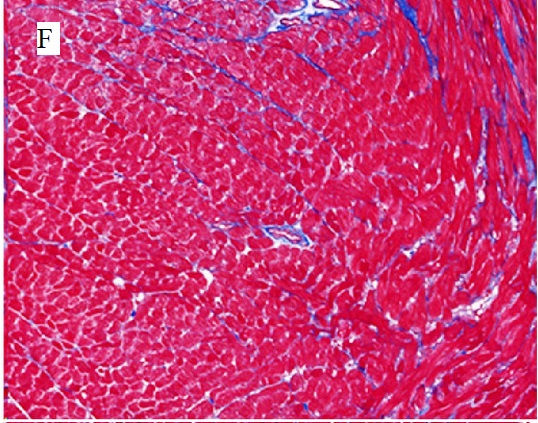

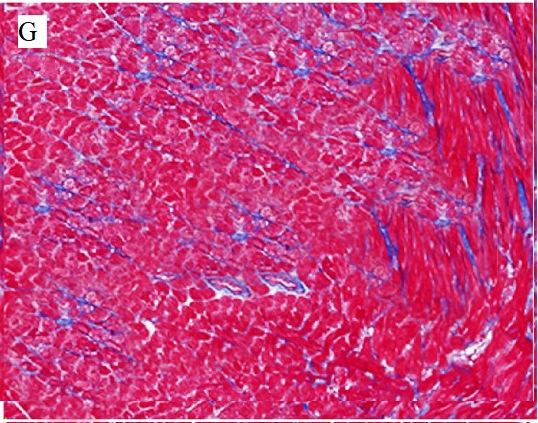

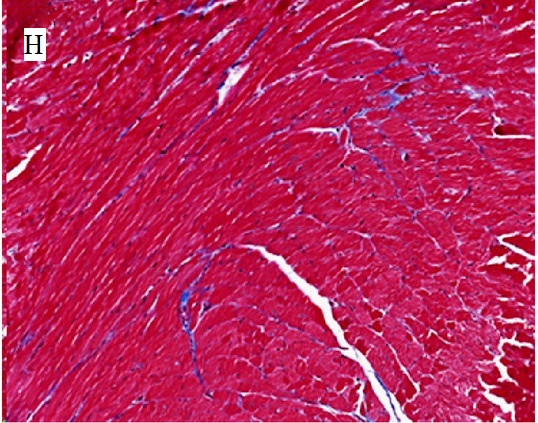


Supplementary figure 6; Masson's trichrome stained sections (40x)of CS (A), S50 (B), S100 (C), S200 (D), CD (E), D50 (F), D100 (G), and D200 (H). The black arrow shows the fibrosis area. CS: healthy W/O AG, S50: healthy with 50 mg/kg AG, S100: healthy with 100 mg/kg AG, S200: healthy with 200 mg/kg AG, CD: diabetic W/O AG, D50: diabetic with 50 mg/kg AG, D100: diabetic with 100 mg/kg AG, D200: diabetic with 200 mg/kg AG. No fibrosis in CS, S50, S100, and S200. On the other hand fibrosis in CD is prominent but rats in groups D50, D100, and D200 showed less fibrosis than the CD. Fibrosis in CD and D100 groups’ rats was significantly more than CS, S50, S100, D50, and D200. Fibrosis in D50 and D200 groups were not significantly more than in healthy groups. There was no significant difference in fibrosis of cardiac tissue in CD and D100 samples.

A


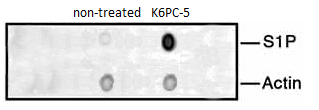


B


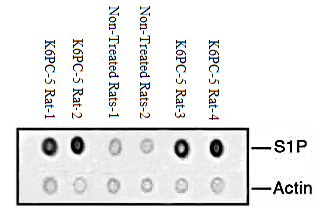


Supplementary figure 7; A sample of western blot gel of non-treated and K6PC-5 (A: blood, B: tissue)


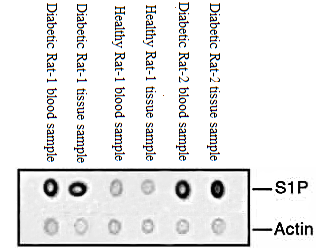


Supplementary figure 8; A sample of western blot gel of Healthy control and Diabetic Rats.

A


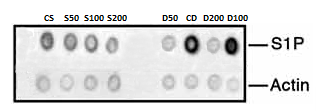


B


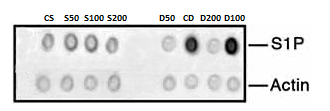


Supplementary figure 9; A: A sample of blood western blot gel of CS, S50, S100, S200, CD, D50, D100, and D200 groups’ rats, B: A sample of tissue western blot gel of CS, S50, S100, S200, CD, D50, D100, and D200 groups’ rats.


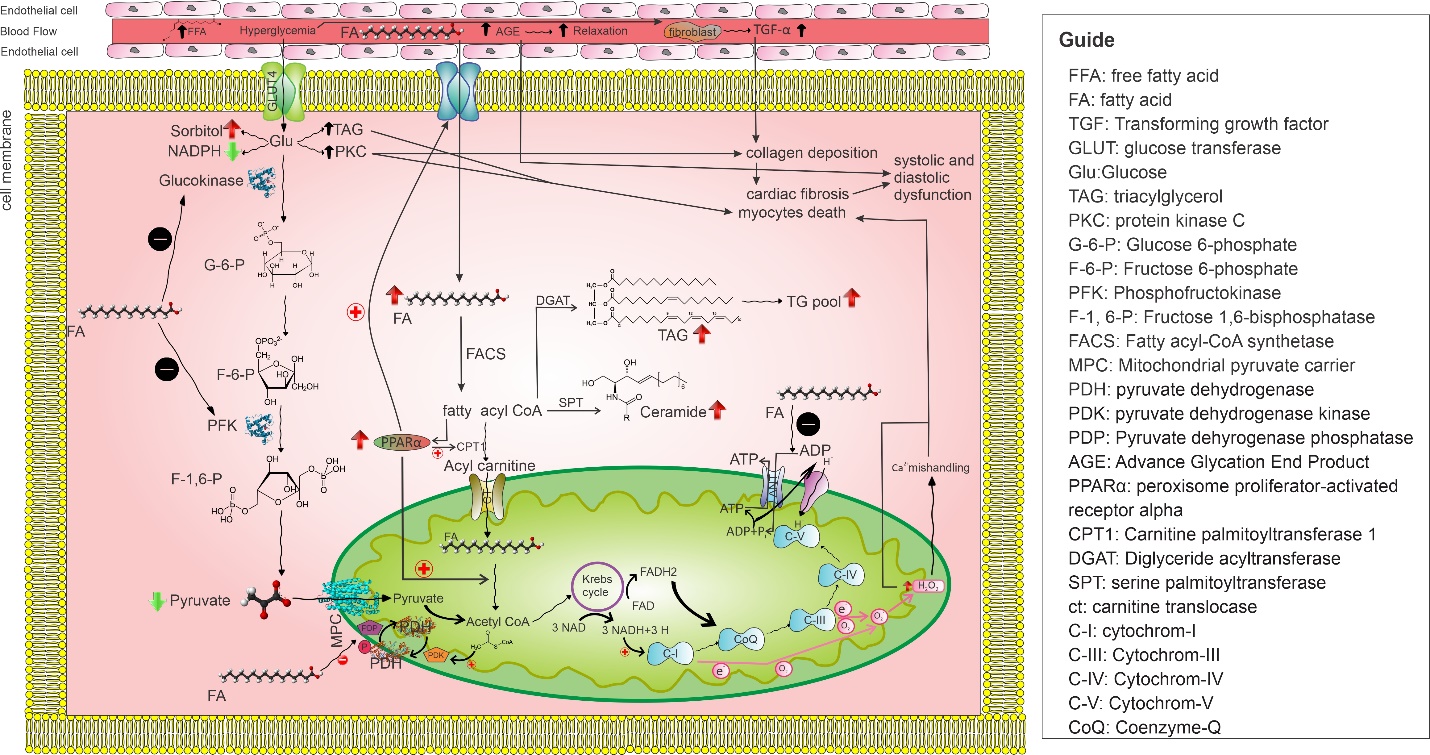


Supplementary figure 10. Pathology pathways of diabetic cardiomyopathy.

FFA consumption leads to the deposition of advanced glycation end products (AGE) which react with proteins, lipids, and nucleic acids. AGE accumulation disrupts cardiac relaxation and induces diastolic dysfunction. Hyperinsulinemia, insulin resistance, and lipotoxicity increase reactive oxygen species (ROS), protein kinase C (PKC), and triacylglycerol (TAG) . Hyperinsulinemia and hyperglycemia lead to calcium imbalance and mitochondrial dysfunction that end up in cell death. Collagen deposition and cardiac fibrosis happen due to transforming growth factor beta (TGF-β) and PKC increase in cardiac fibroblasts. (Supplementary figure 10)


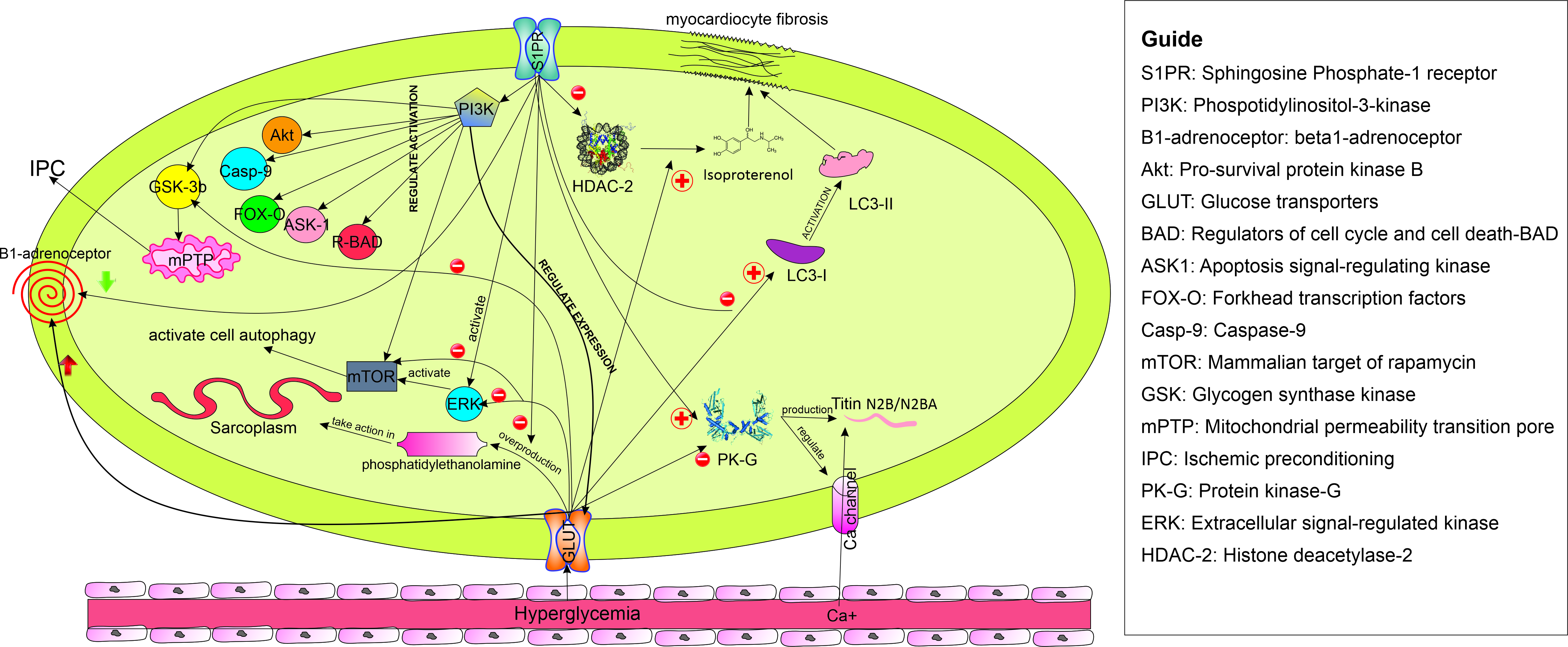


Supplementary figure 11. Mechanism of actions of Sphingosine-1-phosphate through S1P receptors.

S1P is distributed in all tissues and has a critical role in cell life. S1P couples with its receptors (S1PR) to exert its effects. After attaching to S1PR, two kinase pathways start to work. One is Phospotidylinositol-3-kinase (PI3K) and the other is Protein kinase G (PK G). PI3K pathway is continued by other pathways but the most important ones that are crucial in cardiomyocytes living, are Mammalian target of Rapamycin (mTOR) that regulate cell autophagy and Glycogen synthase kinase (GSK)-3b . Glycogen synthase kinase (GSK)-3b exerts its effects by mitochondrial permeability transition pore (mPTP) function. mPTP leads to a function named Ischemic preconditioning (IPC) . IPC is a safety switch for cardiomyocytes that able them to resist ischemic reperfusion (I/R) injuries. The mechanism of this phenomenon is transient episodes of ischemic and reperfusions, which makes cardiomyocytes ready for resistance against tremendous I/R injuries. S1PR also downregulates Β1-adrenoceptor which is a cardioprotector against hyperinsulinemia. An extracellular signal-regulated kinase (ERK) is another pathway that is activated by S1P, it functions through the mTOR path that regulates cell autophagy. There is LC3-I in cardiomyocytes that when it transforms to its active form LC3-II, leads to collagen deposition and fibrosis of the tissue. S1RP inhibits LC3-I activation in cardiomyocytes. Histone deacetylase-2 in cardiomyocytes produces isoproterenol which increases cardiac tissue fibrosis. S1PR inhibits activation of Histone deacetylase-2 (Supplementary figure 11).

***Axillary***

We re-investigated the effect of 100 mg/kg Aminoguanidine to ensure that there was not any bias in the main study. We repeated AG treating study with 12 rats in 3 groups of diabetic control, 100 mg/kg AG treated diabetic, and 200 mg/kg AG treated diabetic rats. Results showed same findings as the main study (Figure-12). We measured S1P level in cardiac tissue of studied rats. 100 mg/kg could not decrease S1P level significantly compare to diabetic control group.


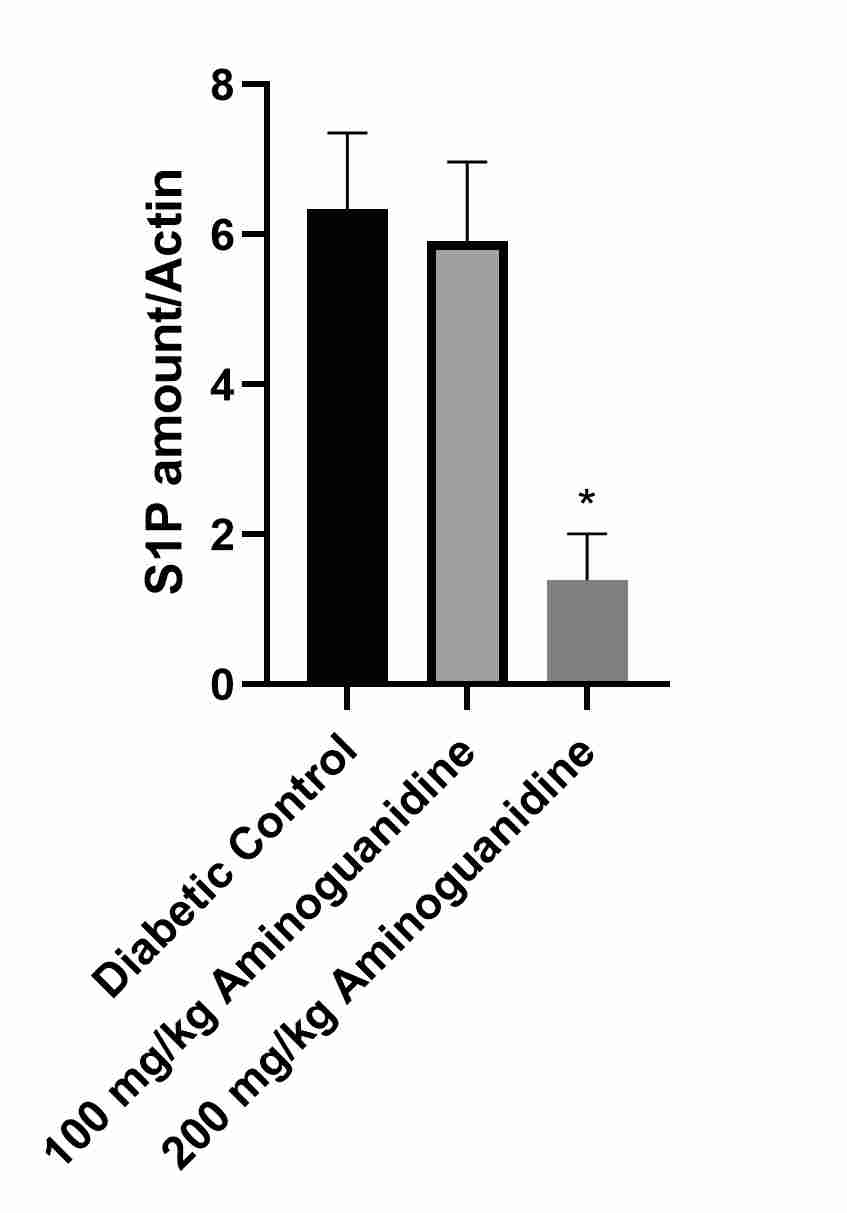


Figure-12, comparison of S1P level/Actin between control diabetic, 100 mg/kg AG, and 200 mg/kg AG groups. * p<0.001
